# The role of damage control surgery in the treatment of perforated colonic diverticulitis: a systematic review and meta-analysis

**DOI:** 10.1007/s00384-020-03784-8

**Published:** 2020-10-22

**Authors:** Roberto Cirocchi, Georgi Popivanov, Marina Konaktchieva, Sonia Chipeva, Guglielmo Tellan, Andrea Mingoli, Mauro Zago, Massimo Chiarugi, Gian Andrea Binda, Reinhold Kafka, Gabriele Anania, Annibale Donini, Riccardo Nascimbeni, Mohammed Edilbe, Sorena Afshar

**Affiliations:** 1 Department of General Surgery, University of Perugia, 06123 Perugia, Italy; 2 Department of Surgery, Military Medical Academy, ul. “Sv. Georgi Sofiyski” 3, 1606 Sofia, Bulgaria; 3 Department of Gastroenterology and Hepatology, Military Medical Academy, ul. “Sv. Georgi Sofiyski” 3, 1606 Sofia, Bulgaria; 4 Department of Statistics and Econometrics, University of National and World Economy, Sofia, Bulgaria; 5 Department of Emergency and Acceptance, Critical Areas and Trauma, “Umberto I” University Hospital, Sapienza University of Rome, 00161 Rome, Italy; 6 Dipartimento di Chirurgia “P. Valdoni”, Sapienza Università di Roma, Viale del Policlinico155, 00161 Rome, Italy; 7 Department of Emergency and Robotic Surgery - A.Manzoni Hospital, Lecco, Italy; 8 Emergency Surgery & Trauma Center, Cisanello University Hospital, 56124 Pisa, Italy; 9 Colorectal Surgery, BioMedical Institute, 16157 Genova, Italy; 10 Department of Visceral, Transplant and Thoracic Surgery, Medical University Innsbruck, Innsbruck, Austria; 11 Department of Medical Science, University of Ferrara, 4121 Ferrara, Italy; 12 Department of Molecular and Translational Medicine, University of Brescia, 25121 Brescia, Italy; 13 North Cumbria Integrated Care NHS Foundation Trust, Carlisle, UK

**Keywords:** Diverticular perforation, Diverticular peritonitis, Damage control surgery

## Abstract

**Introduction:**

Damage control surgery (DCS) is the classic approach to manage severe trauma and has recently also been considered an appropriate approach to the treatment of critically ill patients with severe intra-abdominal sepsis. The purpose of the present review is to evaluate the outcomes following DCS for Hinchey II–IV complicated acute diverticulitis (CAD).

**Methods:**

A comprehensive systematic search was undertaken to identify all randomized clinical trials (RCTs) and observational studies, irrespectively of their size, publication status, and language. Adults who have undergone DCS for CAD Hinchey II, III, or IV were included in this review. DCS is compared with the immediate and definitive surgical treatment in the form of HP, colonic resection, and primary anastomosis (RPA) with or without covering stoma or laparoscopic lavage. We searched the following electronic databases: PubMed MEDLINE, Scopus, and ISI Web of Knowledge. The protocol of this systematic review and meta-analysis was published on Prospero (CRD42020144953).

**Results:**

Nine studies with 318 patients, undergoing DCS, were included. The presence of septic shock at the presentation in the emergency department was heterogeneous, and the weighted mean rate of septic shock across the studies was shown to be 35.1% [95% CI 8.4 to 78.6%]. The majority of the patients had Hinchey III (68.3%) disease. The remainder had either Hinchey IV (28.9%) or Hinchey II (2.8%). Phase I is similarly described in most of the studies as lavage, limited resection with closed blind colonic ends. In a few studies, resection and anastomosis (9.1%) or suture of the perforation site (0.9%) were performed in phase I of DCS. In those patients who underwent DCS, the most common method of temporary abdominal closure (TAC) was the negative pressure wound therapy (NPWT) (97.8%). The RPA was performed in 62.1% [95% CI 40.8 to 83.3%] and the 22.7% [95% CI 15.1 to 30.3%]: 12.8% during phase I and 87.2% during phase III. A covering ileostomy was performed in 6.9% [95% CI 1.5 to 12.2%]. In patients with RPA, the overall leak was 7.3% [95% CI 4.3 to 10.4%] and the major anastomotic leaks were 4.7% [95% CI 2.0 to 7.4%]; the rate of postoperative mortality was estimated to be 9.2% [95% CI 6.0 to 12.4%].

**Conclusions:**

The present meta-analysis revealed an approximately 62.1% weighted rate of achieving GI continuity with the DCS approach to generalized peritonitis in Hinchey III and IV with major leaks of 4.7% and overall mortality of 9.2%. Despite the promising results, we are aware of the limitations related to the significant heterogeneity of inclusion criteria. Importantly, the low rate of reported septic shock may point toward selection bias. Further studies are needed to evaluate the clinical advantages and cost-effectiveness of the DCS approach.

**Electronic supplementary material:**

The online version of this article (10.1007/s00384-020-03784-8) contains supplementary material, which is available to authorized users.

## Introduction

Surgical source control is one of the oldest concepts in the management of intra-abdominal sepsis (IAS): *“Ubi pus ibi evacua”*. In 1889, before the advent of antibiotics, Mikulicz outlined the surgical approach to IAS: early emergency laparotomy, exploration, and washout [1]. During the next century, this became the accepted dogma among emergency surgeons. In 1926, using the same principles, Kirschner demonstrated a decrease in the mortality rate from 90 to 49% [2]. Today, timely surgical intervention, aggressive source control, antibiotics, supportive therapies, and intensive care remain the critical principles in the management of generalized peritonitis due to IAS [3]. Despite many advances, IAS is still associated with high mortality, mainly when associated with septic shock in frail patients [4, 5]. Similar to the management of severe trauma, early surgical treatment of generalized peritonitis is time-dependent and is vital to survival [6]. Damage control surgery (DCS) is the classic approach to managing severe trauma and is defined as an “abbreviated” laparotomy, intensive care unit (ICU) management, and planned reoperation for definitive repair (laparotomy, washout, resection of diseases segment, temporary abdominal closure, stabilization in ICU, reoperation with either end colostomy or anastomosis) [7, 8]. The aim is to avoid the so-called lethal triad of hypothermia, acidosis, and coagulopathy [9, 10]. More recently, DCS has also been considered an appropriate approach to the treatment of critically ill patients with severe IAS [11]. The 2016 World Society of Emergency Surgery (WSES) conference paper stated that “Damage control surgery strategy may be suggested for clinically unstable patients with diverticular peritonitis (severe sepsis/septic shock)” (1B recommendation) [4]. An additional potential benefit of DCS in IAS could be a reduction in the rate of Hartmann’s procedure (HP) and stoma formation [12].

Only a few authors have reported their experience with DCS in the treatment of diffuse peritonitis secondary to complicated acute diverticulitis (CAD) with extreme heterogeneity in the selection criteria and surgical techniques. A systematic review from 2014 reported that DCS was exclusively applied in CAD with septic shock or those requiring vasopressors intraoperatively. However, the authors failed to evaluate whether any physiological parameters (e.g., APACHE and Physiological and Operative Severity Score for the Enumeration of Mortality and Morbidity (POSSUM)) were used to select the patients for DCS [13]. The purpose of the present review is to evaluate the outcomes following DCS for Hinchey III and IV CAD.

## Methods

The Preferred Reporting Items for Systematic Reviews and Meta-analyses (PRISMA) guidelines were followed [14].

### Types of studies

This review included randomized clinical trials (RCTs) and observational studies, both comparative and non-comparative studies, irrespectively of their size, publication status, and language.

### Types of participants

Adults who have undergone DCS for CAD Hinchey II, III, or IV were included in this review.

### Types of interventions

The DCS is compared with the immediate and definitive surgical treatment in the form of HP, colonic resection, and primary anastomosis (RPA) with or without covering stoma or laparoscopic lavage.

### Types of outcome measures

Septic shock, anastomosis, overall leak, major leak, covering stoma, HP, and mortality.

Exclusion criteria were previous reviews, meta-analyses, editorials, letters, and abstracts.

The protocol of this systematic review and meta-analysis was published on Prospero (CRD42020144953).

### Search methods for identification of studies

A comprehensive systematic search was undertaken to identify all relevant studies and articles regardless of language or publication status (published, unpublished, and ongoing). We searched for a wide range of databases and other sources to identify all relevant studies. We searched the following electronic databases with search strategies (SDC 1) without any language or publication restrictions: PubMed MEDLINE (2000 to 13 March 2020); Scopus (2000 to 13 March 2020); and ISI Web of Knowledge (2000 to 13 March 2020).

We searched the following websites of registers of clinical trials: http://www.controlled-trials.com and https://clinicaltrials.gov/ (accessed on 13 March 2020) for ongoing trials on the topic of interest. We manually checked the reference lists of all included studies to identify any additional studies.

### Searching other resources

We performed a search of relevant studies on conference proceedings, theses, and published abstracts reported on Google Scholar.

### Selection of studies

Two authors (RC and GP) reviewed the titles and abstracts of all reports of all the studies identified independently. The full text of studies that possibly fulfill the inclusion criteria was obtained. Any disagreements were resolved by discussion among authors.

### Data extraction and management

Two authors (RC and GP) extracted the data independently. Any disagreements were resolved by a consensus meeting with a third review author (GT). A data extraction form was used to collect information such as trial characteristics (year of publication, country of the study, methodological quality items of the study), participant characteristics, intervention characteristics, comparator characteristics, and outcome characteristics.

### Assessment of risk of bias in included studies

Two authors (RC, GP) assessed the potential risk of bias for each trial. The methodological quality for the RCT was evaluated using the Cochrane “risk of bias” assessment tool for RCTs [15]. RCTs were considered to be at high risk of bias if a high risk was scored in one or more of the critical domains. The comparative non-randomized studies of interventions (NRSI) were evaluated with “Risk Of Bias In Non-randomized Studies of Interventions” (ROBINS-I) scoring system, which is a new tool for assessing the risk of bias [16], and the analysis of non-comparative studies was performed using the MINORS [17].

## Results

**Fig. 1:**
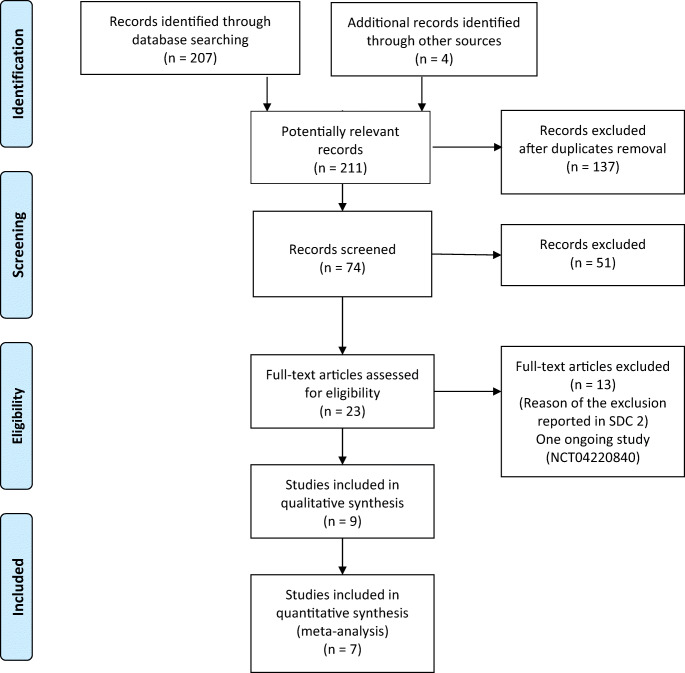
PRISMA flow diagram of study search

The PRISMA flow diagram shows the study search activities performed (Fig. 1). We identified 207 studies using database searches, and four additional records were identified through other sources. After removing duplicates, 74 citations were screened, of which 51 were excluded based on title and abstract. Full texts were obtained and reviewed for the remaining 23 studies. One ongoing study (NCT04220840, first posted at January 7, 2020, with the title “The Damage Control Strategy for the Treatment of Perforated Diverticulitis of the Sigmoid Colon With Diffuse Peritonitis”) [18] and thirteen studies were excluded based on reasons listed in the SDC 2 [19–31]. Nine studies were included in this systematic review and meta-analysis [32–40].

The NCT04220840 is an ongoing study, first posted at January 7, 2020, with the title “The Damage Control Strategy for the Treatment of Perforated Diverticulitis of the Sigmoid Colon With Diffuse Peritonitis.” It is a retrospective multicenter transnational study which intends to compare a large cohort of patients with perforated diverticulitis, treated by DCS or other approach (Hartmann’s resection, lavage, primary anastomosis). Currently, seven centers from Gemany, Austria, and Italy agreed to take part. A positive vote of the ethics committee was obtained in August 2020 and data collection started in September [18]. The study is open for additional centers who are interested to include patients.

## Statistical analysis

A meta-analysis of the rates of seven important indicators, septic shock, anastomosis, overall leak, major leak, covering stoma, HP, and mortality, was conducted. Data from studies were pooled, and weighted mean with a 95% confidence interval (CI) has been calculated for each of the included indicators. Cochrane’s *Q* test and *I*^2^ statistics have been used to define statistical heterogeneity. *I*^2^ statistics measures the proportion of total variation of the respective indicator observed over the studies attributable to differences and specifics between them. In case of significant statistical heterogeneity (when *I*^2^ > 75%), a binary random-effects model for pooling the data about the respective indicator has been employed. Otherwise, when significant statistical heterogeneity has not been presented, a fixed-effects model has been applied for the respective indicator.

## Results

In total, nine studies are found to fulfill the inclusion criteria (Table 1). One RCT [32], three prospective observational studies [36–38], and five retrospective observational studies [33–35, 39, 40]. All studies were performed in Central Europe [33, 34, 37–40]/Italy [35, 36] and published between 2008 and 2020.

**Table 1:** Characteristics of the included studies

Author–year of publication	Nation	Type of the study	Time of the enrolment	Overall patients enrolled in the study	Patients underwent DCS					
					Enrolled in the study	Hinchey II	Hinchey III	Hinchey IV	Septic shock at presentation in ED	In hospital mortality
Kafka 2020	Austria	RCT	2013–2018	21	13	0	9 (69.2%)	4 (30.8%)	NR	1
Gasser 2019	Austria	ROS	2009–2014	78	78	9 (11,5%)	49 (62.8%)	20 (25.7%)	0	15
Brillantino 2019	Italy	POS	2016–2018	30	30	0	17 (56.7%)	13 (43.3%)	1 (3.3%)	1
Tartaglia 2019	Italy	ROS	2011–2017	34	34	0	13 (38.2%)	21 (61.8%)	34 (100%)	4
Sohn 2018	Germany	ROS	2011–2017	74	74	0	60 (81.1%)	14 (18.4%)	16 (22%)	5
Sohn 2016	Germany	ROS	2010–2015	37	19	0	17 (89.5%)	2 (10.5%)	5 (26%)	2
Kafka 2012	Austria	POS	2006–2011	51	51	0	40 (78.4%)	11 (21,6%)	16 (31%)	5
Perathoner 2010	Austria	POS	2006–2008	27	15	0	12 (80%)	3 (20%)	15 (100%)	7
Deenichin 2008	Bulgaria	ROS	2002–2007	6	4	0	0	4 (100%)	4 (100%)	0
Total				358	318	9 (2.8%)	217 (68.3%)	92 (28.9%)	91 (28.6%)	40 (11.2%)

*RCT* randomized control study

*POS* prospective observational study

*ROS* retrospective observational study

*NR* not reported

*ED* emergency department

### Description of the studies

A detailed description of the characteristics of the included patients and the DCS technique used is presented in Table 1 and SDC 3. In total, 358 patients treated between 2002 and 2018 were enrolled in the nine studies, and 318 of these, undergoing DCS, were included in this review. The mean age was between 65 (30–90) years on males and 70.1 (30–92) years on females. The mean BMI was reported only from one study at 28.42 ± 3.3 [35]. Six studies reported an ASA score of 3 or more in 88.4% of patients, and the mean MPI (Mannheim Peritonitis Index) was between 16 and 26.2. The presence of septic shock at the presentation in the emergency department was reported in 91 patients. The majority of the patients had Hinchey III (217 patients, 68.3%) disease. The remainder had either Hinchey IV (92 patients, 28.9%) or Hinchey II (9 patients, 2.8%) disease (Table 1).

### Quality assessment of the included studies

The only included RCT showed an “unclear risk of bias” in random sequence generation and allocation concealment, high risk of bias for blinding (participants, personnel, and outcome assessment), and low risk of bias in attrition and reporting (SDC 4). In the studies of Sohn 2016 [40] and Perathoner [37], the risk of bias of comparative NRSI was respectively low and moderate due to limitations associated with the retrospective design (SDC 5a); the mean MINORS score for the other observational non-comparative NRSI was 9.2 (moderate risk) (SDC 5b).

### Interventions

The three DCS’ phases of each study are described in detail in SDC 6.

Phase I is similarly described in a lot of the studies as lavage, limited resection with blind colonic ends. In few studies, resection and anastomosis [33, 37] or suture of the perforation site [38] were performed in phase I of DCS: RPA in 29 patients (9.1%) and suture of the perforation in 3 patients (0.9%). In those patients who underwent DCS, the most common method of temporary abdominal closure (TAC) was the negative pressure wound therapy (NPWT) (311 patients, 97.8%).

Phases II and III were similarly described in all studies as resuscitation in ICU followed by reoperation after 24–48 h (24–36 h in two studies).

In phase II, the death in ICU was 1.3% (4 patients).

In phase III, the RPA is performed in 197 patients (61.9%) and the HP in 85 patients (26.7%) (Table 2). All patients were evaluated for ongoing peritonitis, and the abdominal wall was definitively closed.

**Table 2:** Surgical treatments performed during DCS

	Patients enrolled	NPWT for open abdomen	Resection and primary anastomosis			Hartmann’s procedure	Suture of perforation	Death before second look
			During the first intervention	During the second look	Covering ileostomy			
Kafka-Ritsch 2020	13	13	0	11	0	1	0	1
Gasser 2019	78	78	26	20	0	30	0	2
Brillantino 2019	30	30	0	24	0	6	0	0
Tartaglia 2019	34	34	0	24	3	10	0	0
Sohn 2018	74	74	0	62	25	12	0	0
Sohn 2016	19	19	0	15	4	4	0	0
Kafka-Ritsch 2012	51	51	0	35	4	12	3	1
Perathoner 2010	15	12	3	6	0	6	0	0
Deenichin 2008	4	0	0	0	0	4	0	0
Total	318	311 (97.8%)	29 (12.8%)	197 (87.2%)	36 (15.9%)	85 (26.7%)	3 (0.9%)	4 (1.3%)
			226 (71%)					

*NPWT* negative pressure wound technique

In sum, the RPA was performed in 226 patients (71%): 29 patients (12.8%) during phase I and 197 patients (87.2%) during phase II. A covering ileostomy is performed in 36 patients (15.9%) who had RPA (Table 2).

The outcomes of interventions of each study are summarized in Table 3 and SDC 7, 8, 9. Reoperation was required in 57.69% of patients with anastomotic leaks. This involves formation of a covering ileostomy in the minor leaks (0%) and colostomy for major leaks (60%) (Table 3).

**Table 3:** Complication in patients underwent colonic resection and primary anastomosis

	Resection and primary anastomosis	Overall leak	Major leak	Minor leak	Reintervention for leak: ileostomy	Reintervention for leak: colostomy
Kafka-Ritsch 2020	11	0	0	0	0	0
Gasser 2019	46	10	NR	NR	NR	NR
Brillantino 2019	24	1	1	0	0	1
Tartaglia 2019	24	NR	1	0	0	1
Sohn 2018	62	8	6	0	3	3
Sohn 2016	15	1	1	0	1	0
Kafka-Ritsch 2012	35	5	3	2	2	3
Perathoner 2010	9	1	1	0	0	1
Deenichin 2008	0	0	0	0	0	0

The overall morbidity rate, according to the Clavien and Dindo classification, was reported only in one study [35]. The most common severe complications were reported in class IIIb (14.7%). Fewer complications were reported in the other classification groups: class IV (5.9%) and IIIa (2.9%) (SDC 7).

The mean length of hospital stays reported in four studies was between 17.5 and 25 days. The ICU length of stay was between 1 and 20 days (SDC 8). The hospital mortality rate was 6.7%; 3 patients died before the third phase of DCS (1.1%) [32, 33].

The rate of incisional hernia was between 23.5% (8/33) [35] and 50% (2/4) [39]. The closure of stoma was performed in 43% of patients: closure of ileostomy in 88% and reversal of colostomy in 22.2% (SDC 9).

The meta-analysis of the rates of seven indicators is performed (Table 4). The study of Deenichin (2008) was excluded due to the low quality [39]. The study of Sohn, published in 2016 [40], was excluded because there is an overlapping in some patients enrolled in the study published in 2018 [34].

**Fig. 2:**
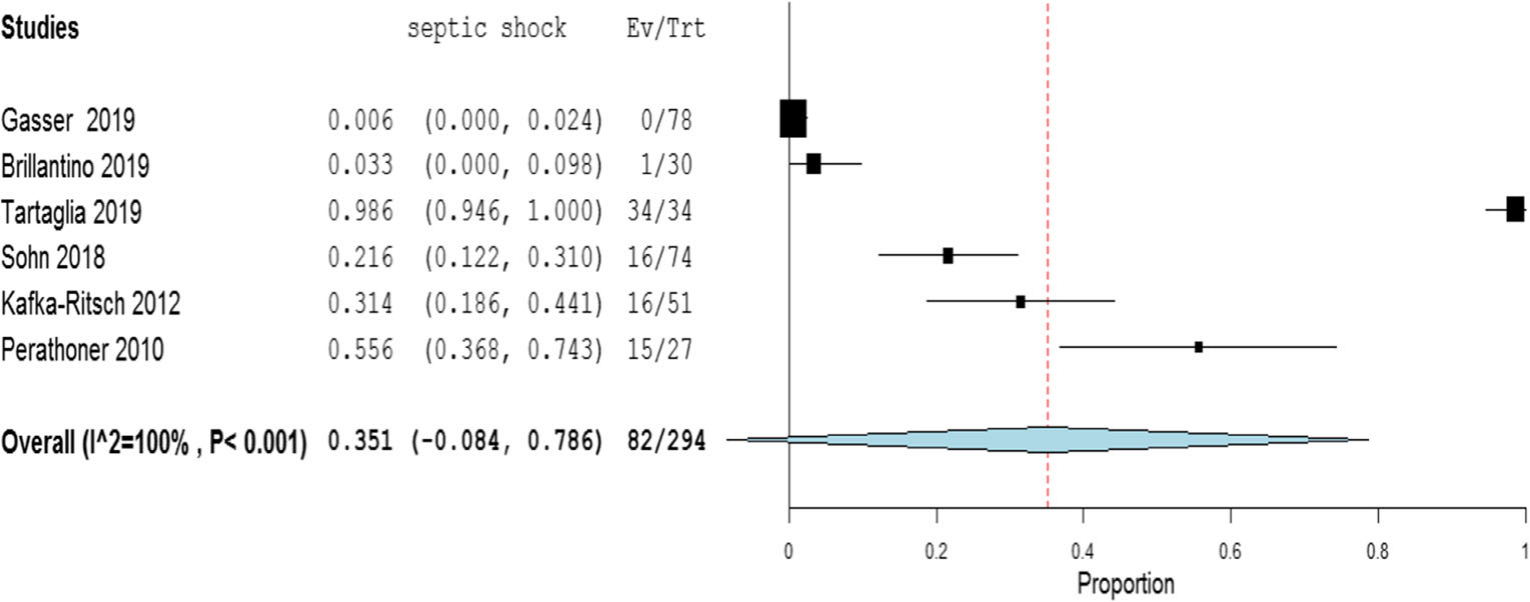
Rate of septic shock in patients underwent DCS

**Fig. 3:**
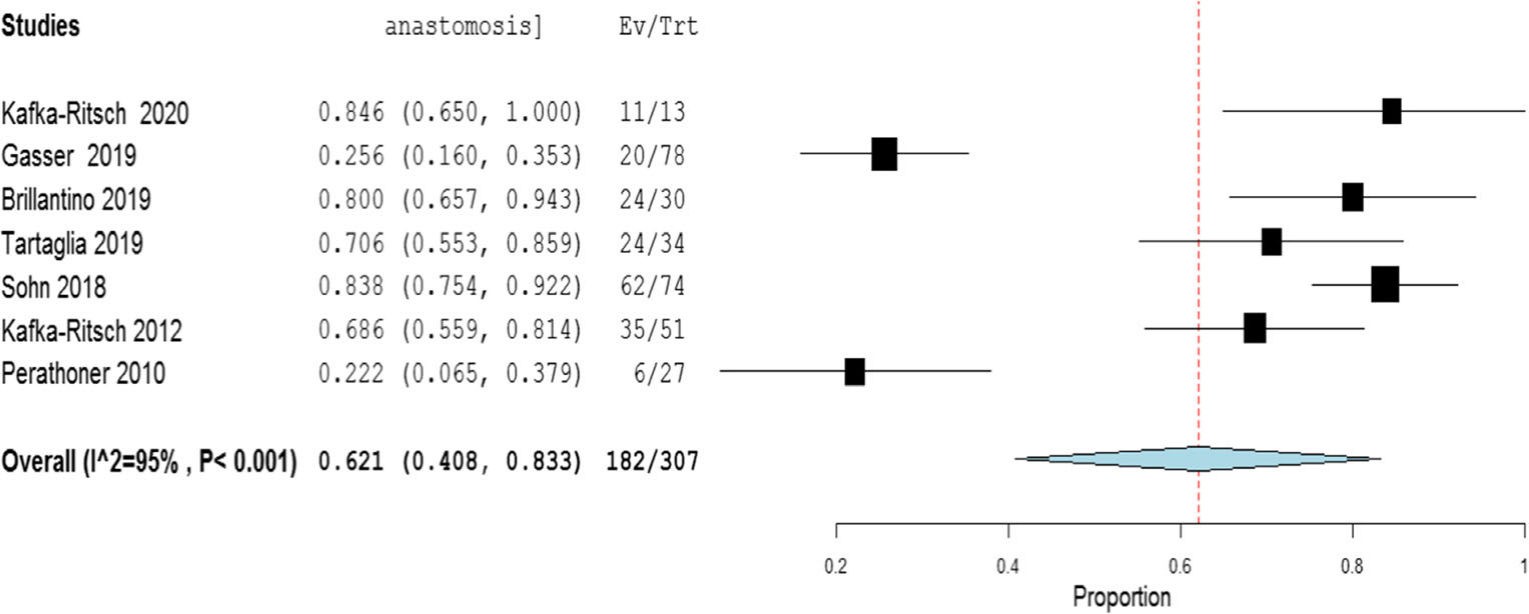
Rate of primary resection and anastomosis in patients underwent DCS

**Fig. 4:**
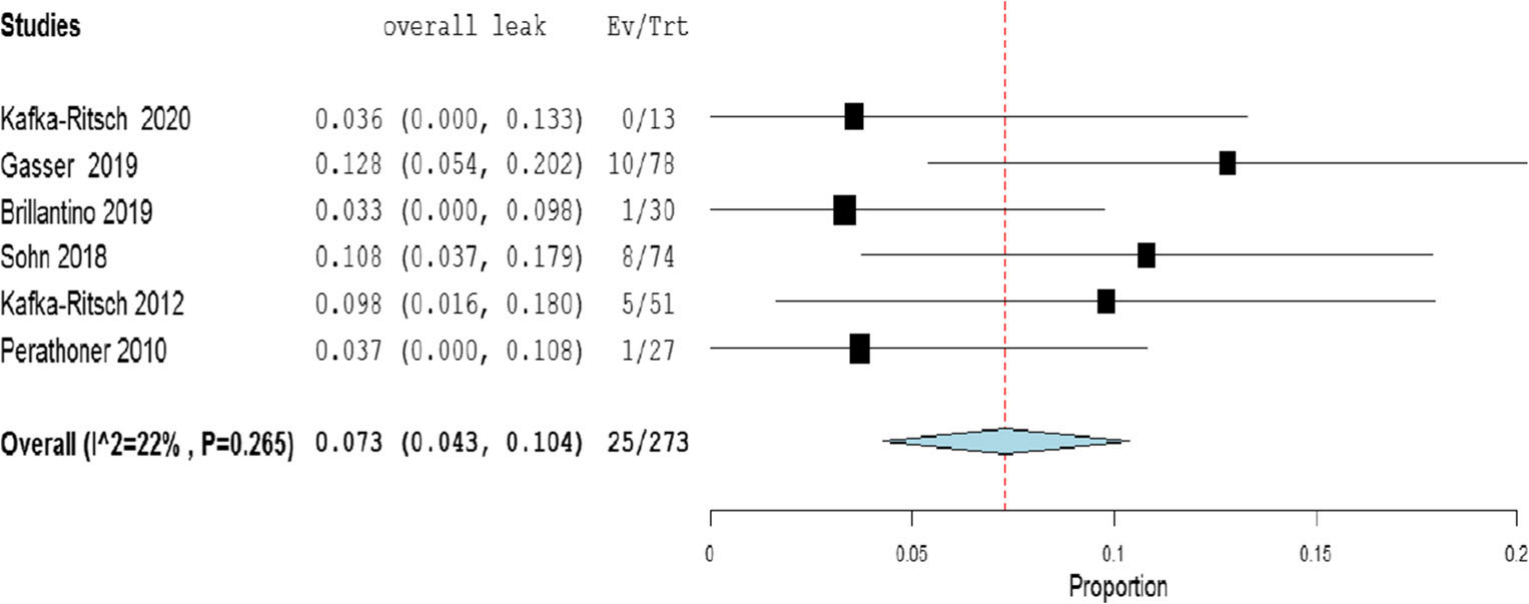
Rate of overall leak in patients with primary colorectal anastomosis who underwent DCS

**Fig. 5:**
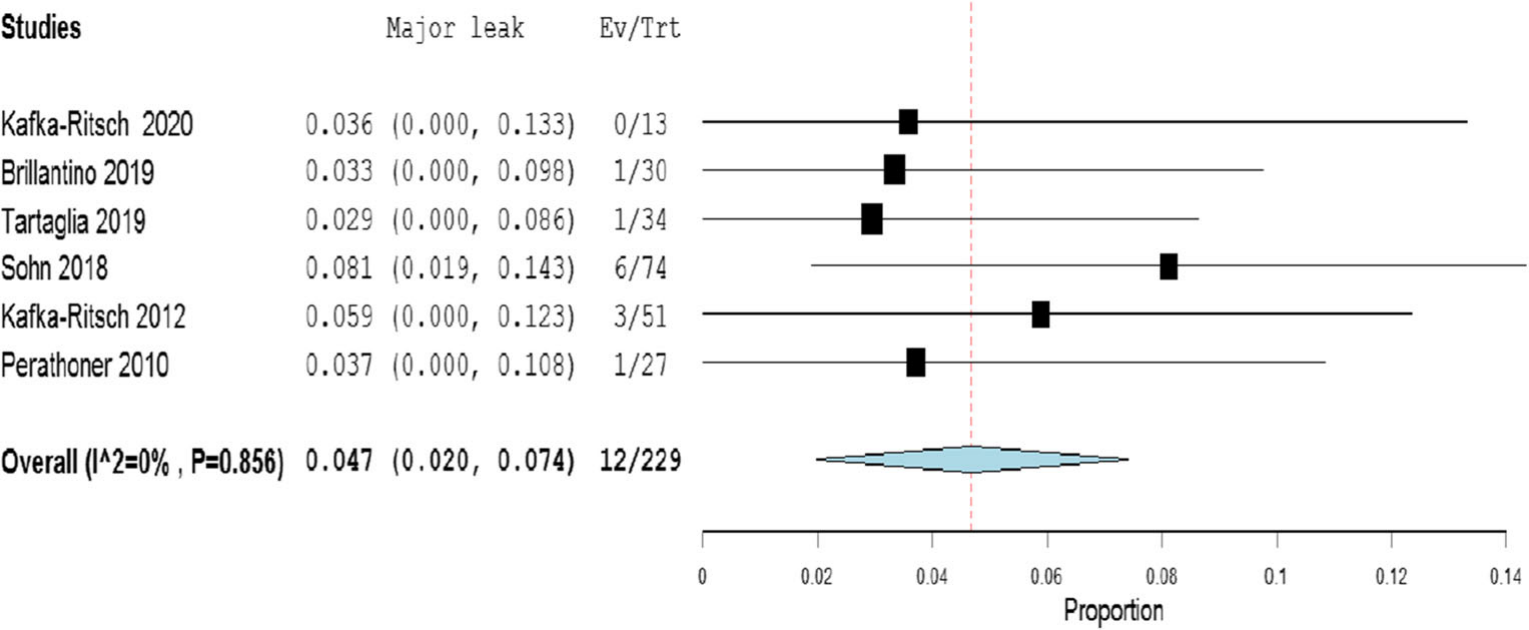
Rate of major leak in patients with primary colorectal anastomosis who underwent DCS

**Fig. 6:**
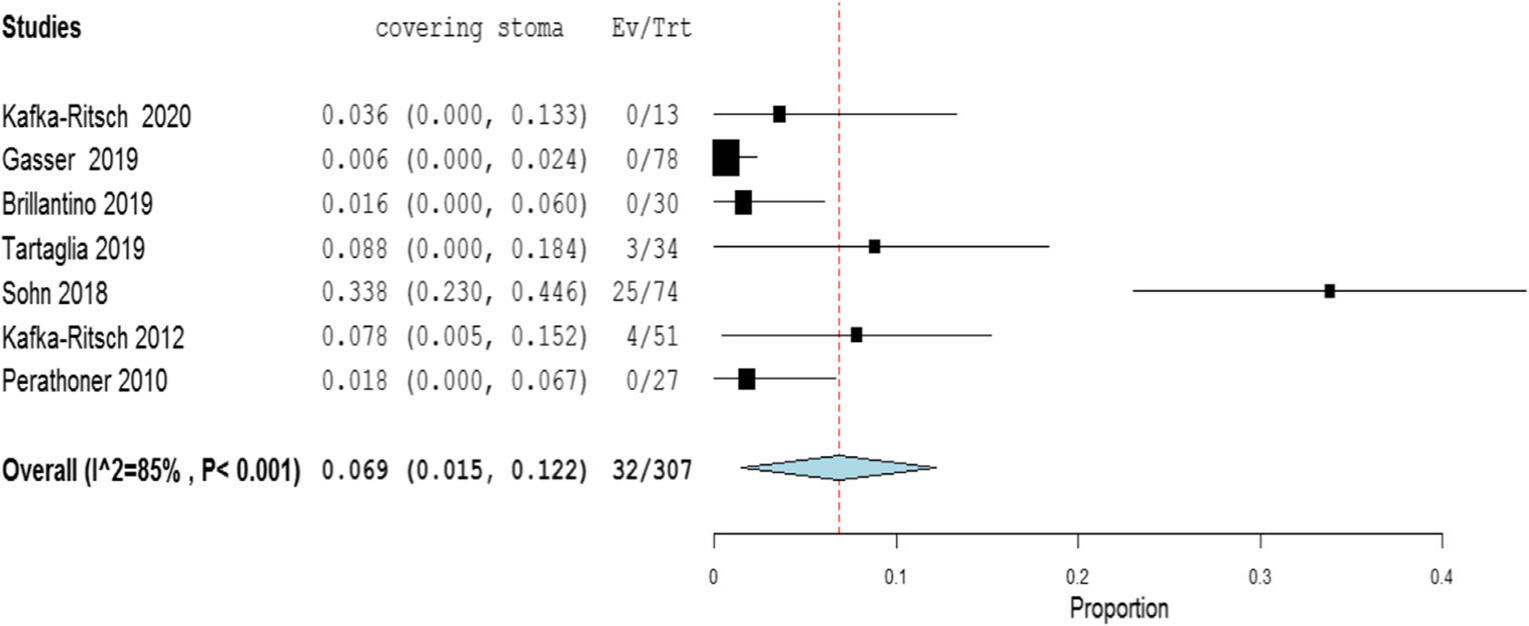
Rate of protective stoma in primary colorectal anastomosis

**Fig 7:**
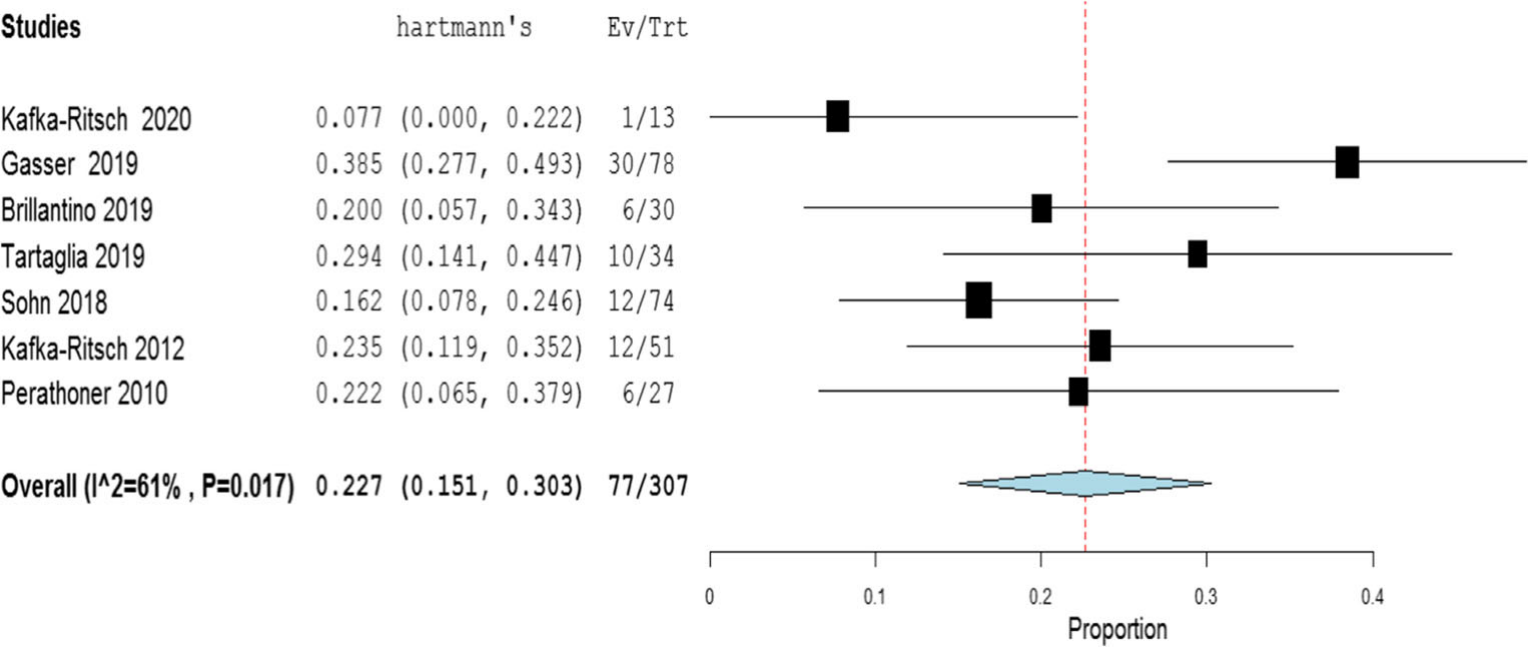
Rate of Hartmann’s in patients underwent DCS

**Fig. 8:**
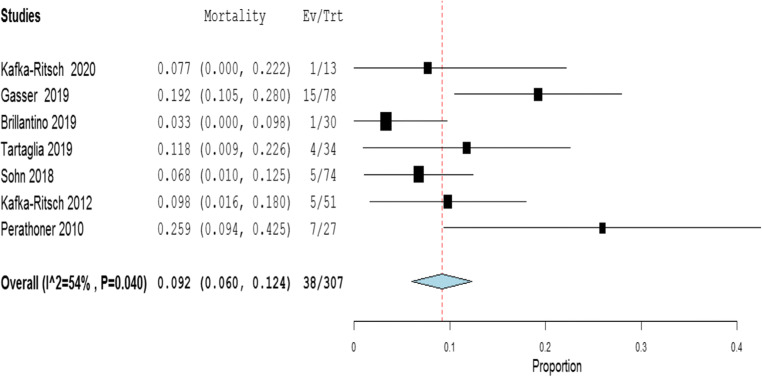
Rate of postoperative mortality in patients underwent DCS

- **Rate of septic shock in patients undergoing DCS**. A high level of heterogeneity was detected across the studies in terms of the rate of septic shock reported. Two studies reported a low rate (< 4%), while one reported a very high rate (> 96%). This high heterogeneity may be driven by variability on the definition of septic shock across centers and countries (Fig. 2). Because of the significant heterogeneity (*I*^2^ = 100%; *P* < 0.001), a binary random-effect model was used. The weighted mean rate of septic shock across the studies was shown to be 35.1% [95% CI 8.4% to 78.6%].
- **Rate of primary resection and anastomosis in patients after DCS**. Meta-analysis using a random-effect model shows a high level of heterogeneity (*I*^2^ = 95%; *P* = < 0.001), and the weighted mean of anastomosis across the studies is 62.1% [95% CI 40.8 to 83.3%] (Fig. 3).
- **Rate of an overall leak in patients with primary colorectal anastomosis after DCS**. Meta-analysis using a fixed-effect model shows a low level of heterogeneity (*I*^2^ = 22%; *P* = 0.265), and the weighted mean rate of overall anastomotic leak is 7.3% [95% CI 4.3 to 10.4%] (Fig. 4).
- **Rate of major anastomotic leaks in patients with primary colorectal anastomosis undergoing DCS**. Meta-analysis using a fixed-effect model shows a low level of heterogeneity (*I*^2^ = 0%; *P* = 0.856), and the weighted mean rate of the major leak is shown to be 4.7% [95% CI 2.0 to 7.4%] (Fig. 5).
- **Rate of protective stoma with primary colorectal anastomosis**. Meta-analysis using a random-effect model shows a high level of heterogeneity (*I*^2^ = 85%; *P* < 0.001), and the weighted mean rate of covering stoma was 6.9% [95% CI 1.5 to 12.2%] (Fig. 6).
- **Rate of HP in patients undergoing DCS**. Meta-analysis using a random-effect model shows a high of heterogeneity (*I*^2^ = 61%; *P* < 0.001), and weighted mean rate of HP is 22.7% [95% CI 15.1 to 30.3%] (Fig. 7).
- **Rate of postoperative mortality in patients undergoing DCS**. Meta-analysis using a fixed-effect model shows a moderate level of heterogeneity (*I*^2^ = 54%; *P* < 0.001), and the weighted mean rate of mortality is estimated to be 9.2% [95% CI 6.0% to 12.4%] (Fig. 8). The results were similar using a random-effect model.

**Table 4:** Summary of the statistical analysis (Fixed-Effect and Random Models)

Outcome	Estimate (weighted mean)	Lower bound – Upper bound (95% Confidance interval)	Std.Error	P value	Heterogeneity		
					Q (df=5)	Het.p-Value	I^2^
Septic shock	0.351	[-0.084; 0.786]	0.222	0.114	2029.6	< 0.001	100%
Anastomosis	0.621	[0.408; 0.833]	0.108	< 0.001	118.1	< 0.001	95%
Overall leak	0.073	[0.043; 0.104]	0.016	< 0.001	6.4	0.265	22%
Major leak	0.047	[0.020; 0.074]	0.014	< 0.001	1.951	0.856	0%
Covering stoma	0.069	[0.015; 0.122]	0.027	0.012	40.3	< 0.001	85%
Hartmann’s procedure	0.227	[0.151; 0.303]	0.039	< 0.001	15.492	0.017	61%
Mortality	0.092	[0.060; 0.124]	0.016	< 0.001	13.169	0.040	54%

## Discussion

Generalized peritonitis secondary to CAD is a relatively uncommon cause of an acute abdomen. According to a recent survey of national databases, the rate varies between 1 and 10% of all admissions for CAD [40]. The surgical treatment requires immediate source control by drainage of the infected fluid collections, debridement, and definitive treatment of the diverticular perforation [41]. Any delay in the treatment of generalized peritonitis with sepsis leads to a high rate of adverse outcomes [42]. Because the number of patients with generalized peritonitis from CAD presenting to each unit is small, no uniform surgical strategy exists. The management of CAD has changed significantly, overtime [43]. In the late nineteenth century, Lockhart-Mummery proposed the abdominal lavage with or without a simple suture of perforated colon and drainage [44]. Later, Mickulicz described the resection with a double-barrelled colostomy [45, 46]. Mayo reported the three-stage procedure (proximal colostomy, resection of the sigmoid colon, closure of the colostomy after few weeks) [47, 48]. Some of the non-resectional approaches were associated with inadequate source control, so the HP (sigmoid resection, burying the rectal stump, and performing end colostomy) became widely used [49]. It remained the standard gold treatment until the 1990s [50, 51] when, in very selected cases, RPA was considered after on-table irrigation of the colon [52]. The subsequent shift toward RPA is based mainly on the growing realization that HP reversal was associated with significant morbidity (55%) and mortality (20%) [53, 54]*.* Additionally, a large number of patients never had closure of their colostomy (48–74%) [53, 55, 56]*.* HP is also associated with postoperative morbidity in up to 52% with a reoperation rate of up to 10%, as well as possible stoma-related complications [57]*.*

A meta-analysis of three RCTs showed that RPA and HP appear to be equivalent in terms of most outcomes of interest, except for a lower intra-abdominal abscess risk after RPA**.** The latter finding needs further investigation as it was not reported in any of the individual trials. However, given the limitations of the included RCTs, no firm conclusion can be drawn as to which is the best surgical option [58]*.*

A protective ileostomy is another attempt to diminish the consequences of anastomotic complications associated with RPA. The ileostomy reversal rate was significantly higher (90% vs. 57%), alongside with lower rate of major complications (0% vs. 20%) and hospital costs [59, 60]. An RCT comparing HP and RPA reported a higher rate of loop ileostomy closure than HP reversal (96% vs. 65%, respectively), with comparable morbidity [61].

The concept of DCS in patients with perforated CAD was first defined in 2010 as surgery with the aim “to enhance patient recovery by means of an initial rapid source control procedure and resuscitation of the patient at the intensive care … the decision on the definitive surgical resolution can be postponed to an elective setting in a hemodynamically stable patient to allow “delayed” reconstruction of bowel continuity” [37, 38]. Accordingly, DCS not only aims at saving lives as in trauma but also at reducing the rate of HP. This concept was also included in the 2015 WSES position paper on the role of open abdomen in managing severe abdominal sepsis. The low rates of septic shock and Hinchey IV peritonitis in some reports raised concerns about potential selection bias [62]. The present analysis revealed a weighted rate of the septic shock of approximately 35.1%, with substantial heterogeneity of the included studies, and 28.9% of the cases had Hinchey IV peritonitis. Only Tartaglia [35], Perathoner [37], and Deenichin [39] reported the presence of septic shock in all cases of their series, differently Brillantino et al. in only 1 of 30 [36] and Sohn in 14 of 74 [34]. No patients with septic shock were reported in the series from Gasser et al. [33].

The restoration of GI continuity was achieved in 62,1% of the cases, which can be interpreted as a significant success when compared to > 50% of patients not having their stoma reversed after HP, although this is not the primary aim of the DCS approach [63, 64]. As in the trauma scenario, the primary objective is to avoid the lethal triad of hypothermia, acidosis, and coagulopathy. Success depends not only on the approach employed or the precise surgical technique but also on sound judgment, accurate assessment of the disease and the general status of the patient, and timely intervention.

The overall and major leak rates were 7.3% and 4.7%, respectively, which are similar to the rates reported in the literature [58, 59, 65]. The defunctioning loop ileostomy can be a useful tactic to “protect” anastomoses or to treat a minor leak. Surprisingly, the weighted rate of covering stoma in the present meta-analysis was very low (6.9%). Similarly to Oberkofler et al. and Bridoux et al., the rate of ileostomy closure in the present study was higher than HP reversal (88% vs. 22%). The weighted mortality of 9.2% is also comparable with the literature data [58].

The limitations of the present study include the small sample size, the moderate quality of the observational studies, the only one RCT with a high risk of bias. Another limitation is the lack of subgroup analysis, which could not be performed due to the shortage of data. A selection bias toward a higher rate of RPA after DCS is possible because only 35.1% of the cases were in septic shock and 28.9% with Hinchey IV peritonitis. The success of the DCS approach is highly dependent on the indications and the correct selection of the candidates for RPA [33]. Inappropriate application of DCS can be dangerous due to complications. The improper use of DCS is associated with an increased risk for bowel perforation, sepsis, multiorgan failure, prolonged hospital stays, and mortality [66]. In the included studies, no complications related to open abdomen (entero-atmospheric fistula or frozen abdomen) were reported. Potential explanations are inadequate follow-up or improvements in the commercially available NPWT systems [67]. DCS can also pose a significant burden on hospital resources and increases the cost of the treatment [68]. None of the studies included in this review reported a cost analysis, which is another limitation.

## Conclusions

The present meta-analysis revealed an approximately 62.1% weighted rate of achieving GI continuity with the DCS approach to generalized peritonitis secondary to CAD with major leaks of 4.7% and overall mortality of 9.2%. Despite the promising results, we are aware of the limitations related to the significant heterogeneity of inclusion criteria. Importantly, the low rate of reported septic shock and the lack of reported definition may point toward selection bias. Based on the available data, we suggest a tailored approach according to the severity of the disease and condition of the patient.

Further studies are needed to evaluate the clinical advantages and cost-effectiveness of the DCS approach and to help identify patients suitable for RPA and the role of covering ileostomy. The RCT from Kafka-Ritsch, recently published, is well planned, but recruitment was difficult, and only 13 patients were enrolled within a relatively long period [32]. To overcome these issues, we are waiting the results of ongoing study NCT04220840 (“The Damage Control Strategy for the Treatment of Perforated Diverticulitis of the Sigmoid Colon With Diffuse Peritonitis”) [18] and recommend a prospectively, randomized multicenter trial with support from international surgical society.

## Electronic supplementary material

ESM 1 (DOCX 14 kb).

ESM 2 (DOCX 15 kb).

ESM 3 (DOCX 19 kb).

ESM 4 (DOCX 17 kb).

ESM 5 (DOCX 299 kb).

ESM 6 (DOCX 18 kb).

ESM 7 (DOCX 15 kb).

ESM 8 (DOCX 13 kb).

ESM 9 (DOCX 16 kb).

ESM 10 (DOCX 16 kb).
