# Supplementary material for: The role of damage control surgery in the treatment of perforated colonic diverticulitis: a systematic review and meta-analysis
Source: Int J Colorectal Dis. 2020 Oct 22;36(5):867–79. doi: 10.1007/s00384-020-03784-8 (PMC8026449; doi:10.1007/s00384-020-03784-8)
Supplement: Supplementary file 1 — (DOCX 14 kb). [file 384_2020_3784_MOESM1_ESM.docx]

Appendix A: **Search strategies.**

PubMed

1. damage[All Fields] AND (“prevention and control”[Subheading] OR (“prevention”[All Fields] AND “control”[All Fields]) OR “prevention and control”[All Fields] OR “control”[All Fields] OR “control groups”[MeSH Terms] OR (“control”[All Fields] AND “groups”[All Fields]) OR “control groups”[All Fields]) AND (“surgery”[Subheading] OR “surgery”[All Fields] OR “surgical procedures, operative”[MeSH Terms] OR (“surgical”[All Fields] AND “procedures”[All Fields] AND “operative”[All Fields]) OR “operative surgical procedures”[All Fields] OR “surgery”[All Fields] OR “general surgery”[MeSH Terms] OR (“general”[All Fields] AND “surgery”[All Fields]) OR “general surgery”[All Fields]) AND (“diverticulitis”[MeSH Terms] OR “diverticulitis”[All Fields])
2. damage[All Fields] AND (“prevention and control”[Subheading] OR (“prevention”[All Fields] AND “control”[All Fields]) OR “prevention and control”[All Fields] OR “control”[All Fields] OR “control groups”[MeSH Terms] OR (“control”[All Fields] AND “groups”[All Fields]) OR “control groups”[All Fields]) AND (“laparotomy”[MeSH Terms] OR “laparotomy”[All Fields]) AND (“diverticulitis”[MeSH Terms] OR “diverticulitis”[All Fields])
3. (“colon, sigmoid”[MeSH Terms] OR (“colon”[All Fields] AND “sigmoid”[All Fields]) OR “sigmoid colon”[All Fields] OR “sigmoid”[All Fields]) AND (“diverticulitis”[MeSH Terms] OR “diverticulitis”[All Fields]) AND damage[All Fields] AND (“prevention and control”[Subheading] OR (“prevention”[All Fields] AND “control”[All Fields]) OR “prevention and control”[All Fields] OR “control”[All Fields] OR “control groups”[MeSH Terms] OR (“control”[All Fields] AND “groups”[All Fields]) OR “control groups”[All Fields]) AND (“surgery”[Subheading] OR “surgery”[All Fields] OR “surgical procedures, operative”[MeSH Terms] OR (“surgical”[All Fields] AND “procedures”[All Fields] AND “operative”[All Fields]) OR “operative surgical procedures”[All Fields] OR “surgery”[All Fields] OR “general surgery”[MeSH Terms] OR (“general”[All Fields] AND “surgery”[All Fields]) OR “general surgery”[All Fields])feculent [All Fields] AND (“peritonitis”[MeSH Terms] OR “peritonitis”[All Fields])

Scopus

 (*damage*  AND *control*  AND *surgery*  AND *diverticulitis* ) )  AND  ( *damage*  AND *control*  AND *surgery*  AND *laparotomy*  AND *diverticulitis* )

Web of Science

( *damage*  AND *control*  AND *surgery*  AND *diverticulitis* ) )  AND  ( ( ( *damage*  AND *control*  AND *surgery*  AND *laparotomy*  AND *diverticulitis* ) )  AND  ( *damage*  AND *control*  AND *surgery*  AND *sigmoid*  AND *colon*  AND *diverticulitis* ) )  AND  ( *damage*  AND *control*  AND *surgery*  AND *sigmoid*  AND *colon*  AND *diverticulitis* )
