# Supplementary material for: The role of damage control surgery in the treatment of perforated colonic diverticulitis: a systematic review and meta-analysis
Source: Int J Colorectal Dis. 2020 Oct 22;36(5):867–79. doi: 10.1007/s00384-020-03784-8 (PMC8026449; doi:10.1007/s00384-020-03784-8)
Supplement: Supplementary file 2 — (DOCX 15 kb). [file 384_2020_3784_MOESM2_ESM.docx]

Appendix B. **Excluded studies and reason for exclusion.**

| **Author** | **Year of publication** | **Reason for exclusion** |
| --- | --- | --- |
| Rosenzweig et al. | 2020 | Miscellaneous of colonic perforation (acute diverticulitis, chronic diverticular disease, colon cancer, enterocolitis, ulcerative colitis, volvulus, et al.) |
| Tachezy et al | 2019 | Review |
| Sohn et al | 2018 | Letter |
| Zizzo et al. | 2018 | Letter |
| Ceresoli et al. | 2018 | Review |
| Fiocchi et al | 2015 | Not reported data about the patients underwent DCS |
| Kwon et al | 2013 | Review |
| Moore et al | 2013 | Review |
| Ferrada et al. | 2013 | Review |
| Liang et al. | 2012 | The technique reported is not the classic DCS |
| Moore et al. | 2012 | Review |
| Nystrom et al. | 2010 | Review |
| Vermeulen et al. | 2010 | Review |
