# Supplementary material for: The role of damage control surgery in the treatment of perforated colonic diverticulitis: a systematic review and meta-analysis
Source: Int J Colorectal Dis. 2020 Oct 22;36(5):867–79. doi: 10.1007/s00384-020-03784-8 (PMC8026449; doi:10.1007/s00384-020-03784-8)
Supplement: Supplementary file 3 — (DOCX 19 kb). [file 384_2020_3784_MOESM3_ESM.docx]

SDC 3. **Characteristics of patients underwent DCS.**

| **Study** | **Registration of the protocol** | **Declared conflict of interest** | **Mean age (years) ± SD** | **Gender (M)** | **Gender (F)** | **Mean BMI ± SD** | **ASA score =/> 3** | **MPI (± SD)** |
| --- | --- | --- | --- | --- | --- | --- | --- | --- |
| **Kafka-Ritsch 2020** | ClinicalTrials.gov: NCT04034407 | None | 67.2 (45-92) | 7 | 6 | NR | NR | 26 (12-37) |
| **Gasser 2019** | NR  NR | None | 65 (30-90)* | 38 | 40 | NR | NR | 22 (0-33)* |
|  |  | None | 67 (43-86)^ |  |  |  |  | 22 (11-39)^ |
| **Brillantino 2019** | NR | None | 68.5 (35-84) | 12 | 18 | NR | 30 | 26.2 (12-40) |
| **Tartaglia 2019** | NR | None | 66.9 ± 12.7 | 15 | 19 | 28.42 ± 3.3 | 22 | 25.12 ± 6.28 |
| **Sohn 2018** | NR | None | 67 (30–92) | 34 | 40 | NR | 58 | 22.4 (6–42) |
| **Sohn 2016** | NR | None | NR | 6 | 13 | NR | 15 | 16 |
| **Kafka-Ritsch 2012** | NR | None | NR | 23 | 28 | NR | 51 | NR |
| **Perathoner 2010** | NR | None | 67 ± 9.6 | 7 | 8 | NR | 15 | 23.9 ± 5.4 |
| **Deenichin 2008** | NR | None | NR | NR | NR | NR | NR | NR |

NR: not reported

NC: not calculable

*laparotomy using the ABThera™ (KCI, Wien, Austria) abdominal NPT device

^laparotomy using the SuprasorbVR CNP (Lohmann & Rauscher, Wien, Austria) abdominal NPT device
