# Supplementary figures and images for: The role of damage control surgery in the treatment of perforated colonic diverticulitis: a systematic review and meta-analysis
Source: Int J Colorectal Dis. 2020 Oct 22;36(5):867–79. doi: 10.1007/s00384-020-03784-8 (PMC8026449; doi:10.1007/s00384-020-03784-8)

SDC 4: **Summary assessment of risk of bias for the included randomised control trial.**


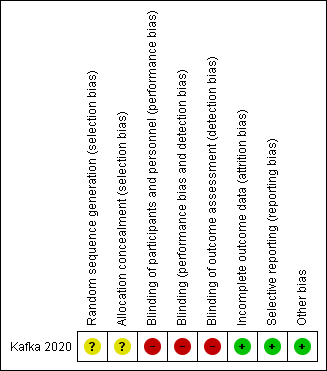

Supplement: Supplementary file 4 — (DOCX 17 kb). [file 384_2020_3784_MOESM4_ESM.docx]
