# Supplementary material for: The role of damage control surgery in the treatment of perforated colonic diverticulitis: a systematic review and meta-analysis
Source: Int J Colorectal Dis. 2020 Oct 22;36(5):867–79. doi: 10.1007/s00384-020-03784-8 (PMC8026449; doi:10.1007/s00384-020-03784-8)

SDC 5a. **The risk of bias assessment of included papers using ROBINS-I tool for non-randomized comparative studies**

**
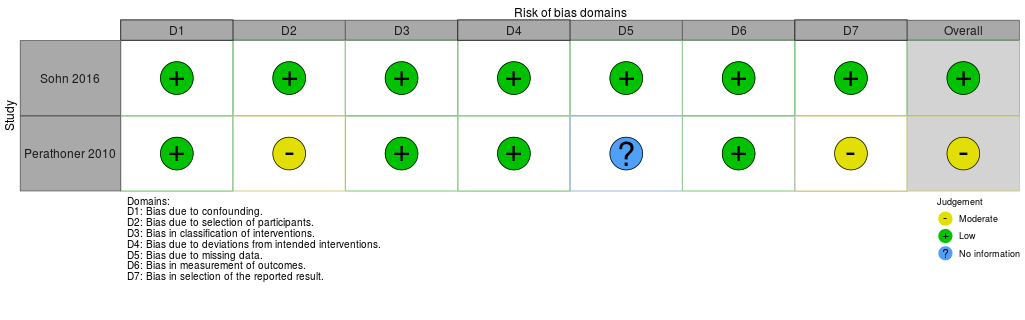
**


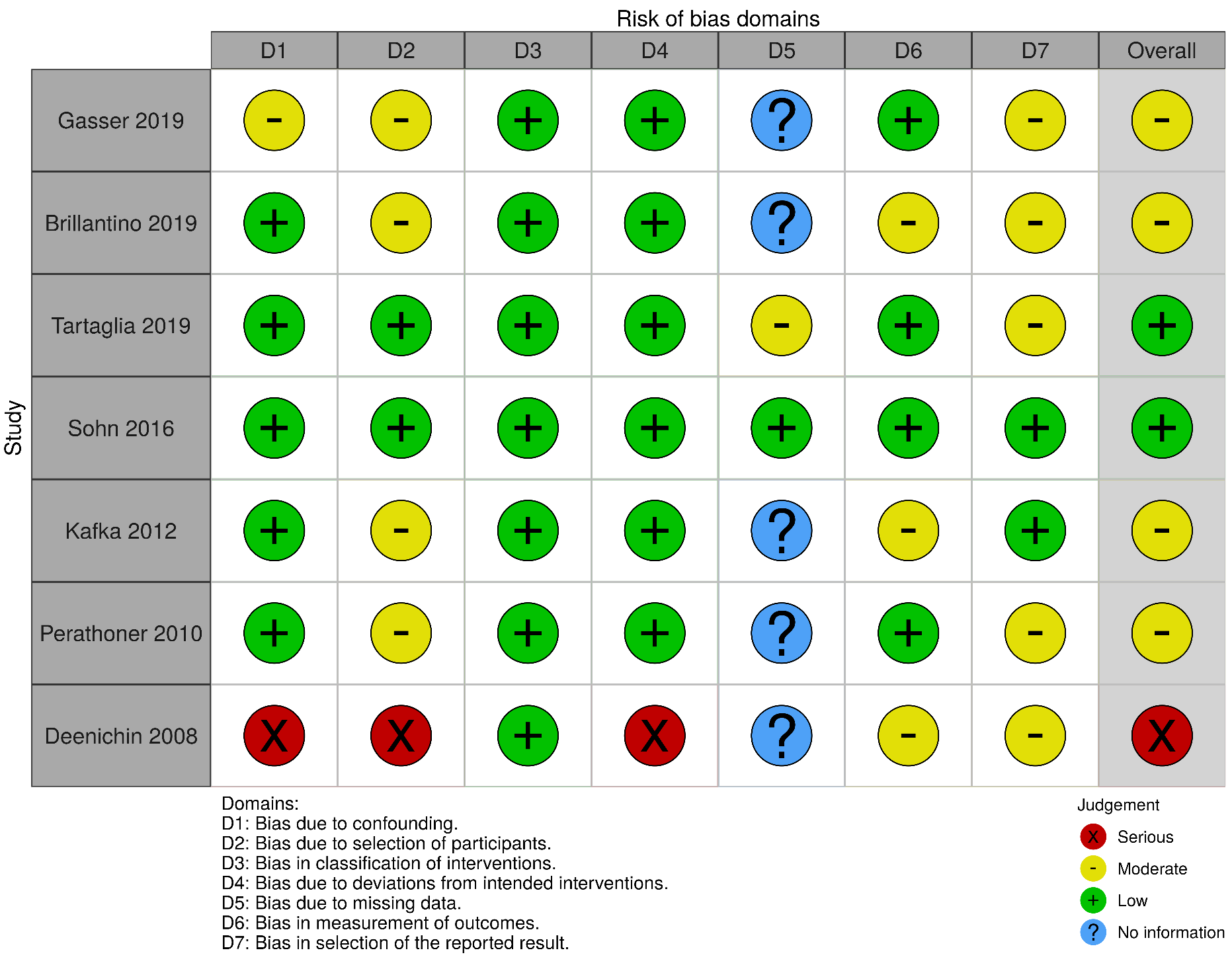

Supplement: Supplementary file 5 — (DOCX 299 kb). [file 384_2020_3784_MOESM5_ESM.docx]
