# Supplementary material for: The role of damage control surgery in the treatment of perforated colonic diverticulitis: a systematic review and meta-analysis
Source: Int J Colorectal Dis. 2020 Oct 22;36(5):867–79. doi: 10.1007/s00384-020-03784-8 (PMC8026449; doi:10.1007/s00384-020-03784-8)
Supplement: Supplementary file 6 — (DOCX 18 kb). [file 384_2020_3784_MOESM6_ESM.docx]

SDC 5b. **Methodological assessment of the included observational studies according to the Methodological Items for Nonrandomized Studies score (MINORS).**

|  | **Gasser**  **2019** | **Brillantino 2019** | **Tartaglia 2019** | **Sohn 2018** | **Kafka-Ritsch 2012** | **Deenichin**  **2008** |
| --- | --- | --- | --- | --- | --- | --- |
| A clearly stated aim | 2 | 2 | 2 | 2 | 2 | 2 |
| Inclusion of consecutive patients | 2 | 1 | 0 | 2 | 2 | 0 |
| Prospective collection of data | 0 | 2 | 0 | 0 | 2 | 0 |
| Endpoints appropriate to the aim of the study (intention to treat) | 1 | 2 | 2 | 1 | 2 | 0 |
| Unbiased assessment of the study endpoint (blinding) | 0 | 0 | 0 | 0 | 0 | 0 |
| Follow-up period appropriate to the aim of the study | 2 | 2 | 2 | 2 | 2 | 2 |
| Loss to follow-up <5% | 2 | 2 | 2 | 2 | 2 | 2 |
| Total | 9 | 11 | 8 | 9 | 12 | 6 |

Item Score: 0 (not reported); 1 (reported but inadequate); 2 (reported and adequate).
