# Supplementary material for: The role of damage control surgery in the treatment of perforated colonic diverticulitis: a systematic review and meta-analysis
Source: Int J Colorectal Dis. 2020 Oct 22;36(5):867–79. doi: 10.1007/s00384-020-03784-8 (PMC8026449; doi:10.1007/s00384-020-03784-8)
Supplement: Supplementary file 7 — (DOCX 15 kb). [file 384_2020_3784_MOESM7_ESM.docx]

SDC 6. **Phases of DCS**.

|  | **I° Phase DCS**  **(Initial emergency operation)** | **II° Phase DCS (ICU stay)** | **III° Phase DCS**  **(Reoperation)** |
| --- | --- | --- | --- |
| **Kafka-Ritsch 2020** | Preliminary source control: lavage, limited bowel resection (sigmoid resection with blind colonic ends). NPWT. | Resuscitation in ICU | After 24 - 48 hrs a planned second look |
| **Gasser**  **2019** | Preliminary source control: lavage, limited bowel resection (sigmoid resection with blind colonic ends or anastomosis or stoma). Successively anastomosis, no anastomosis and no ostomy or ostomy. NPWT. | Resuscitation in ICU | After 24 - 48 hrs a planned second look |
| **Brillantino 2019** | Preliminary source control: lavage, limited bowel resection (sigmoid resection with blind colonic ends). NPWT. | Resuscitation in ICU | After 24 - 48 hrs a planned second look |
| **Tartaglia**  **2019** | Preliminary source control: lavage, limited bowel resection (sigmoid resection with blind colonic ends). NPWT. | Resuscitation in ICU | After 24 - 48 hrs a planned second look |
| **Sohn**  **2018 -2016** | Preliminary source control: lavage, limited bowel resection (sigmoid resection with blind colonic ends). NPWT. | Resuscitation in ICU | After 24 - 48 hrs a planned second look |
| **Kafka-Ritsch 2012** | Preliminary source control: lavage, limited bowel resection (sigmoid resection with blind colonic ends) or closure of the perforation site. NPWT. | Resuscitation in ICU | After 24 - 48 hrs a planned second look |
| **Perathoner 2010** | Preliminary source control: lavage, limited bowel resection (sigmoid resection with blind colonic ends or anastomosis or stoma) or closure of the perforation site. NPWT. | Resuscitation in ICU | After 24 - 36 hrs a planned second look |
| **Deenichin**  **2008** | Preliminary source control: lavage, limited bowel resection (sigmoid resection with blind colonic ends). TAC without NPWT. | Resuscitation in ICU | After 24 - 36 hrs a planned second look |

NPWT (Negative Pressure Wound Therapy)

TAC (Temporary Abdominal Closure)
