# Supplementary material for: The role of damage control surgery in the treatment of perforated colonic diverticulitis: a systematic review and meta-analysis
Source: Int J Colorectal Dis. 2020 Oct 22;36(5):867–79. doi: 10.1007/s00384-020-03784-8 (PMC8026449; doi:10.1007/s00384-020-03784-8)
Supplement: Supplementary file 8 — (DOCX 13 kb). [file 384_2020_3784_MOESM8_ESM.docx]

SDC 7:  **Overall morbidity rate according to the Clavien and Dindo classification.**

|  | **Patients enrolled (number)** | **I** | **II** | **IIIa** | **IIIb** | **IV** |
| --- | --- | --- | --- | --- | --- | --- |
| **Tartaglia 2019** | 34 | 0 | 6 | 1 | 5 | 2 |
