# Supplementary material for: The role of damage control surgery in the treatment of perforated colonic diverticulitis: a systematic review and meta-analysis
Source: Int J Colorectal Dis. 2020 Oct 22;36(5):867–79. doi: 10.1007/s00384-020-03784-8 (PMC8026449; doi:10.1007/s00384-020-03784-8)
Supplement: Supplementary file 9 — (DOCX 16 kb). [file 384_2020_3784_MOESM9_ESM.docx]

SDC 8: **Post-operative hospital stays**

|  | **Mean length of hospital stay (± SD) (days)** | **Mean ICU length of stay (days)** |
| --- | --- | --- |
| **Kafka-Ritsch 2020** | 17.5 (12-43) | 2 (1-20) |
| **Gasser 2019** | 22 (1-126) | 6 (0-55) |
|  | 25 (8-72) | 6 (2-46) |
| **Brillantino 2019** | 18 (12-62) | NR |
| **Tartaglia 2019** | 21.9 ± 16.24 | 14 ± 13.5 |
| **Sohn 2018** | 18.5 (3–66) | 22 (3–66) |
| **Sohn 2016** | 18 (3–37) | 2 (0–17) |
| **Kafka-Ritsch 2012** | NR | NR |
| **Perathoner 2010** | NR | NR |
| **Deenichin 2008** | NR | NR |
