# Supplementary material for: The role of damage control surgery in the treatment of perforated colonic diverticulitis: a systematic review and meta-analysis
Source: Int J Colorectal Dis. 2020 Oct 22;36(5):867–79. doi: 10.1007/s00384-020-03784-8 (PMC8026449; doi:10.1007/s00384-020-03784-8)
Supplement: Supplementary file 10 — (DOCX 16 kb). [file 384_2020_3784_MOESM10_ESM.docx]

SDC 9. **Late post-operative outcomes.**

|  | **Closure ileostomy** | **Hartmann’s reversal** |
| --- | --- | --- |
| **Kafka-Ritsch 2020** | 0 | 0 |
| **Gasser 2019** | NR | NR |
| **Brillantino 2019** | 0 | 0 |
| **Tartaglia 2019** | 3 | 0 |
| **Sohn 2018** | NR | NR |
| **Sohn 2016** | 5 | 0 |
| **Kafka-Ritsch 2012** | 5 | 8 |
| **Perathoner 2010** | 0 | 2 |
| **Deenichin 2008** | NR | NR |
| **Total** | 13 (88%) | 10 (22%) |
